# Occurrence of Ticks and Tick-Borne Pathogens During Warm Winter—A Snapshot from Central Europe

**DOI:** 10.3390/pathogens14040326

**Published:** 2025-03-28

**Authors:** Weronika Buczek, Alicja Buczek, Marek Asman, Agnieszka Borzęcka-Sapko, Ewelina Minciel, Jadwiga Grzeszczak, Katarzyna Bartosik

**Affiliations:** 1 Department of Biology and Parasitology, Chair of Pharmacology and Biology, Faculty of Health Sciences, Medical University of Lublin, Radziwiłłowska 11 St., 20-080 Lublin, Poland; alicja.buczek@umlub.pl; 2 Department of Medical and Molecular Biology, Faculty of Medical Sciences in Zabrze, Medical University of Silesia in Katowice, Jordana 19 St., 41-808 Zabrze, Poland; masman@sum.edu.pl; 3 Med-Laser Non-Public Health Care Centre, 20-406 Lublin, Poland; agnieszka.borzecka@op.pl; 4 Department of Medical Chemistry, Faculty of Pharmacy, Medical Biotechnology and Laboratory Medicine, Pomeranian Medical University in Szczecin, 70-204 Szczecin, Poland; ewelina.m555@wp.pl (E.M.); jadwiga.grzeszczak@pum.edu.pl (J.G.)

**Keywords:** *Ixodes ricinus*, *Dermacentor reticulatus*, winter activity, tick-borne pathogens, sexual behavior of ticks, tick anomalies

## Abstract

Background: Climate warming and anthropogenic environmental changes impact the spread of ticks and tick-borne pathogens (TBPs). This study investigated the occurrence of ticks and the risk of TBPs infection in urban and rural recreational areas in Eastern Poland at record-high temperatures in winter. Methods: Ticks were collected from vegetation using the flagging method. Various types of polymerase chain reactions were applied to detect *Borrelia burgdorferi* s.l., *Anaplasma phagocytophilum*, *Rickettsia* spp., and *Babesia* spp. in the studied ticks. Results: 268 ticks were sampled in the four urban/suburban and one rural sites, including 78 *Ixodes ricinus* specimens and 190 *Dermacentor reticulatus* ticks. Of the ticks, 49.19% were infected by at least one TBP, including 41.03% of *I. ricinus* and 63.04% of *D. reticulatus* specimens. Co-infections with TBPs that involved only *I. ricinus* were recorded in 6.41% of these ticks. Conclusions: The study indicates that hosts are exposed to tick attacks and TBPs infection in Central Europe at high temperatures in winter. The high activity of ticks may increase the incidence of tick-borne diseases in humans and companion animals. The record’s importance indicates that practical preventive measures against TBPs should be taken due to weather conditions rather than the season.

## 1. Introduction

*Ixodes ricinus* (Linnaeus, 1758) and *Dermacentor reticulatus* (Fabricius, 1794) (Ixodida: Ixodidae) are ticks with the broadest occurrence range and the greatest diversity of transmitted pathogen species in Europe. They are vectors of human infectious diseases, such as Lyme borreliosis, Spotted Fever rickettsioses, and tick-borne encephalitis, as well as diseases affecting farm animals and companion animals, such as babesiosis [1,2].

The importance of *I. ricinus* and *D. reticulatus* in the epidemiology of tick-borne diseases (TBDs) is associated with their extraordinary ability to adapt to environmental conditions and the biological features of both species, such as the three-host development cycle and the wide range of hosts for larvae, nymphs and adults, which contribute to pathogen dissemination in nature. *Ixodes ricinus* is the most common tick feeding on humans in Western, Central, and Northern Europe [3,4,5,6,7,8,9]. All developmental stages of this tick can feed on humans, with nymphs and females infesting these hosts most frequently [8]. *Dermacentor reticulatus* is a less frequent parasite of humans; nevertheless, for several years, its adults have been increasingly found feeding on humans [5,7,8,10,11]. Both tick species equally frequently infest companion animals present in their habitats [12,13,14,15]. On companion animals, *D. reticulatus* ticks can be transferred to human households, where they find suitable conditions for development. It has been confirmed that adult *D. reticulatus* cease feeding on dogs and infest the skin of dog owners [11].

The spread of *I. ricinus* and *D. reticulatus* ticks is promoted by global warming and environmental changes induced by human activities, such as urbanization, habitat fragmentation, deforestation, and reforestation [16,17,18,19,20,21,22,23]. A measurable effect of these changes is the expansion of these ticks into urban and suburban areas [11,24,25,26,27,28,29,30]. Notably, a high dynamics of tick spread has been observed in recent years in the case of *D. reticulatus*, which used to be primarily associated with meadows, marshes, riverbeds and coastal forests, but now it also occurs in agricultural areas and habitats with highly degraded hydrological and landscape conditions [11,15,22,30,31,32].

In Central Europe, the seasonal tick activity begins after the winter diapause [33,34]. In the natural environment, the threshold temperature values that stimulate *I. ricinus* nymphs and adults to quest for hosts in Central and Northern Europe are above 5 °C [35,36] or above 7 °C [37], depending on the region. The questing activity of *I. ricinus* nymphs is significantly intensified at temperatures >10 °C [38]. Adult *D. reticulatus* are already active at air temperatures ranging from 0 °C to 2.5 °C [39] (Buczek A. own observations), and 4 °C [40].

The seasonal activity of *I. ricinus* usually lasts from early spring to autumn and has two peaks: higher in May–July and lower in September. The latter peak may not occur when, along with the increase in summer temperatures, low humidity remains in the habitat, which is unfavorable for the development of this species and favors desiccation of host-seeking ticks [16,24,25,41,42]. Single-peak activity may be observed in urban and suburban areas of large agglomerations, which may be associated with the environmental conditions (the diurnal course of vapor pressure) prevailing in urban heat islands [25].

The peak of the activity of *I. ricinus* nymphs is usually shifted in time by approx. 3–4 weeks compared to that of adults and is the highest in spring [43]. The seasonal pattern of questing in *D. reticulatus* adults is always bimodal, with a spring peak from April to May and an autumn peak from September to November [22,40,41,44,45]. Depending on the habitat and/or weather conditions, the abundance of ticks in both peaks may vary. The duration of the activity of *D. reticulatus* adults is correlated with weather conditions. It most often begins in March and lasts until November or even December at higher temperatures and the absence of snow cover. Less is known about the rhythms of the activity of *D. reticulatus* juvenile stages. Depending on the region and ambient temperature, the peak activity of *D. reticulatus* nymphs is recorded in July or August [46,47,48].

Given the dynamically changing environmental conditions influencing the spread and dynamics of tick population activity, there is a need for monitoring the presence of these vectors and associated tick-borne pathogens (TBPs) in various types of habitats and regions, especially in urbanized areas where humans and animals can be infested by questing ticks during leisure and work in different seasons of the year.

The present study focused on the investigation of tick occurrence in urban and rural recreational areas in Eastern Poland during an exceptionally warm winter month and the assessment of the infestation risk during this period of the year.

## 2. Materials and Methods

### 2.1. Study Area

The study was conducted in Lublin (51°15′0″ N 22°34′0″ E), which is one of the largest cities in Poland in terms of population, and its surroundings. Lublin occupies a topographically diverse area of 147.5 km^2^. Its western part has a varied topography with numerous deep dry valleys, hills, and loess ravines. In turn, the eastern part of the city has a flat surface. Within the administrative boundaries of Lublin, there are many green areas (parks, several forests, including Dąbrowa and Stary Gaj forests) and Zalew Zemborzycki Lake. There are also large forest complexes in the city’s surroundings.

The city has a different climate from that of non-urban areas and other urbanized areas. The minimum and maximum temperatures are higher in the city center than in its non-urban areas; this indicates the occurrence of an urban heat island in Lublin [49]. The climate of the city is influenced by its location on the Bystrzyca River with two tributaries: the Czerniejówka and Czechówka Rivers, which ensures good air flow. The green areas along streets act as corridors through which air from non-urban areas enters the center. The topography of the city also plays an important role, as it determines and diversifies its climate.

Lublin and its surroundings are located in the humid continental climate zone. The average annual air temperature is 7.3 °C. July with an average temperature of approx. +18.2 °C is the warmest month, while February, with an average temperature of approx. −4.0 °C is the coldest. The summer and vegetation periods last 100–110 and 210–220 days, respectively. The mean annual precipitation is approximately 560 mm. The snow cover persists from 70 to 90 days [50]. In the coldest winter months (January and February), the mean diurnal, nocturnal, and daily temperatures are −0.3 °C, −4.5 °C, and −2.4 °C, as well as 1.3 °C, −4.2 °C, and −1.5 °C, respectively. The precipitation sum in January is 38.7 mm, with an average of 16.9 days with precipitation and an average of 39.8 sunshine hours. February is characterized by the precipitation sum of 33.7 mm, on average 16.4 days with precipitation, and the mean sunshine duration of 71.2 h [51].

**Figure 1:**
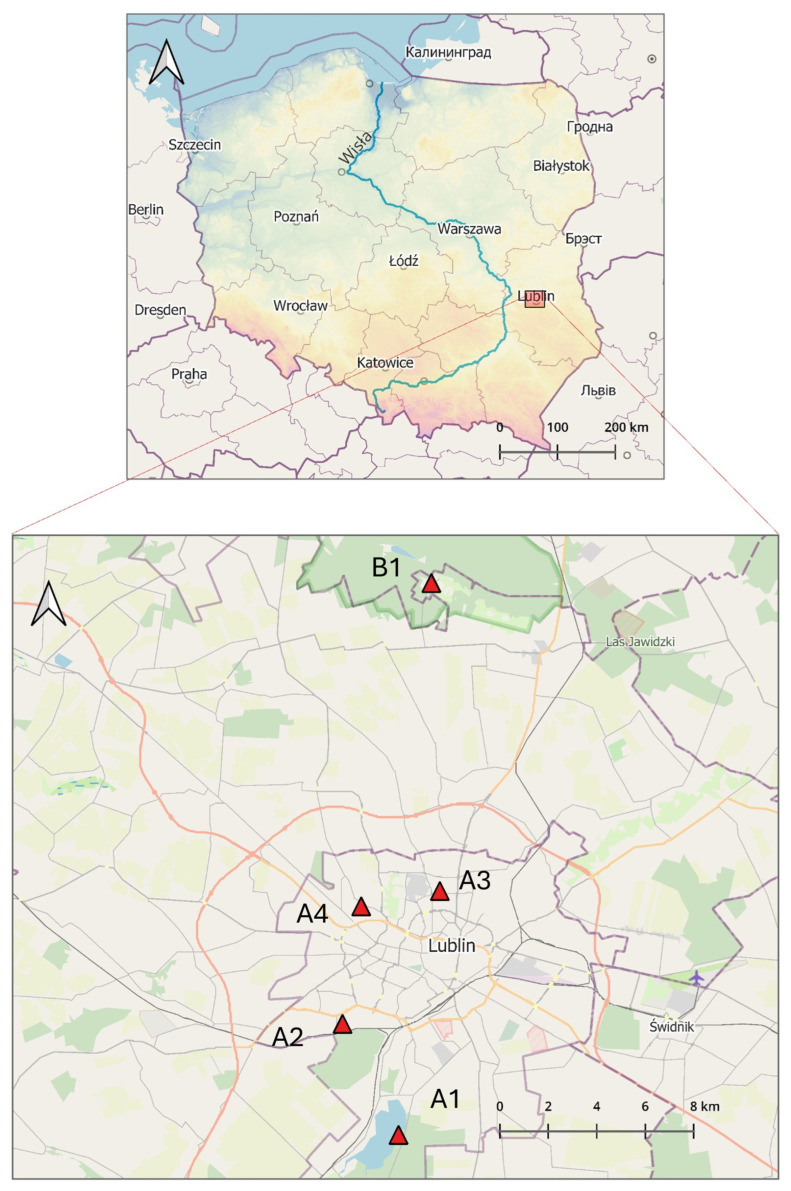
Geographical location of the tick sampling sites within the area of Lublin in Eastern Poland (51°15′0″ N 22°34′0″ E) (prepared by Marcin Wasilewski, marcinwasilewski.eu on the basis of the OpenStreetMap; © authors OpenStreetMap).

The tick collection sites selected for the study included diverse habitats frequently visited by people for recreational purposes (Figure 1 and Figure S1):

A1. Zalew Zemborzycki Lake—a retention and recreational water body on the Bystrzyca River surrounded by mixed Dąbrowa forest with a predominance of European red pine (*Pinus silvestris*) and English oak (*Quercus robur*), followed by an admixture of silver birch (*Betula pendula*), European aspen (*Populus tremula*) and common alder (*Alnus glutinosa*) [52].

A2. Stary Gaj forest complex with dominant deciduous tree species: *Q. robur*, *B. pendula*, *Carpinus betulus*, and small-leaved lime (*Tilia cordata*) and coniferous trees: *P. silvestris*, Norway spruce (*Picea abies*), and common juniper (*Juniperus communis*) [52].

A3. green area (area of agricultural wasteland) in the middle of the Czechów housing estate (area c.a. 2.2087 ha), a remnant of agricultural cultivation that continued until the early 2000s.

A4. garden of a private property (area c.a. 147 m^2^) in the strict city center overgrown by the common snowdrop (*Galanthus nivalis*) and perennial ryegrass (*Lolium perenne*) located in the vicinity of a park with numerous species of shrubs, perennials, and trees represented by valuable old specimens, with the greatest natural value of white poplar (*Populus alba*) and silver lime (*Tilia tomentosa*).

B1. site in a rural area located in Nowy Staw village in the Kozłowiecki Landscape Park located 14,4 km from Lublin, with a mixed forest dominated by European red pine (*Pinus silvestris*) and oaks (*Quercus robur*, *Quercus petraea*) and comprising patches with a close-to-natural composition, meadows, clearings, marshes, peat bogs, and artificial ponds [53].

Forest complexes and fields within city limits favor the occurrence of animals typical of these habitats. Thus, the collection sites can accommodate a wide range of animals that serve as potential hosts for both tick species. In winter, wild animals migrating from forests, such as hares (*Lepus europaeus*), foxes (*Vulpes vulpes*), wild boars (*Sus scrofa*), and roe deer (*Capreolus capreolus*), can be found within the city limits [52].

### 2.2. Tick Collection

Ticks were collected in five localities from 25 to 27 February 2024, between 10.00 a.m. and 3.00 p.m. As part of the reconnaissance work, ticks were collected at Zalew Zemborzycki Lake (A1) also in January 2024, when the weather was sunny and there was no snow cover (4 January 2024). Questing ticks were collected in this habitat using the flagging method [54,55,56]. It consists of sweeping vegetation with a white flannel flag (surface 1 m^2^) over plants and low bushes. Ticks were collected by one person at the majority of the sites for 1 h. Specimens attached to the flag were transferred to sterile polypropylene containers. At one location (private garden, A4), the collection was only conducted for 15 min due to the small size of the site. During each collection round, the temperature and relative humidity on the ground surface were recorded with an accuracy and resolution of 1 °C/0.1 °C and 3.5%/0.1% RH, respectively, using the Temperature/Humidity Datalogger Reed R6030 (Reed Instruments, Wilmington, NC, USA). In the laboratory, the ticks were preserved in 70% ethanol, viewed using a stereo microscope SZX16 (Olympus, Hamburg, Germany), and identified to the species, developmental stage, and sex based on the identification keys [54,55].

The scale proposed by Supergan and Karbowiak [57] was used to determine the risk of host attacks by ticks in each collection site. As specified by these authors, the risk of tick infestation is very high, high, and middle when the number of ticks collected by one person for 1 h is >50 specimens, 26–50 specimens, and 11–25 specimens, respectively.

### 2.3. Molecular Studies

The DNA from individual tick specimens was extracted using the ammonia method [58], and the DNA concentration was measured spectrophotometrically in the NanoPhotometer PEARL (Implen, Munich, Germany) at the 260/280 nm wavelength. Next, all the samples were frozen and stored at −20 °C for further molecular analyses. To detect *Borrelia burgdorferi* sensu lato in the ticks with the real-time PCR method, a Borrelia qPCR detection Kit (EURx, Gdańsk, Poland) was used according to the manufacturer’s protocol. In turn, two pairs of primers specific to the 16S rRNA gene were used to detect *A. phagocytophilum* [59]. *Babesia* spp. and *Rickettsia* spp. were detected in the ticks by single PCR. Primers specific to the 18S rRNA and gltA genes were used to detect these pathogens in the studied ticks [60,61]. Table S1 shows the pairs of specific primers used to detect selected TBPs. The amplification products of PCR and nested PCR were separated electrophoretically in 2% ethidium bromide-stained agarose gels and visualized under ultraviolet light in a device for agarose gel analysis (Vilber Lourmat, Collegien, France). The presence of amplification products with a size of 932 base pairs [bp] and 546 bp for *A. phagocytophilum*, 620 bp for *Babesia* spp., and 381 bp for *Rickettsia* spp. was considered positive.

### 2.4. Statistical Analysis

The chi-square test was applied to test differences in the number of ticks collected in urban and rural locations. Differences with the *p*-value ≤ 0.05 were considered statistically significant.

## 3. Results

As early as January 2024, single active *I. ricinus* specimens (2 females and 3 males) were recorded in the A1 site at a temperature of 7.2 °C and 68.3% RH. Numerous ticks were observed in the study area in February 2024 at ground surface temperatures ranging from 13.9 °C to 24.1 °C and relative humidity from 40.5% to 72.5% RH. In this winter month, 268 ticks were collected in the four urban/suburban and one rural sites, including 78 *I. ricinus* specimens (42 females, 23 males, and 13 nymphs) and 190 *D. reticulatus* ticks (124 females, 61 males, and 5 nymphs) (Table 1). Both tick species were found in most of these sites. The small garden located next to the large park in the city center was the locality where only *I. ricinus* specimens were found. Adults, especially females, dominated the tick species collected from the entire study area and the individual sites. The female:male sex ratio was similar in both tick species studied: F:M = 2.0 in *D. reticulatus* and F:M = 1.8 in *I. ricinus*. Active *I. ricinus* nymphs were found in all the urban localities, while *D. reticulatus* nymphs were only noted in the rural locality.

Within the city limits, from 23 to 39 ticks (*I. ricinus* and *D. reticulatus*) were collected during 1 h in each of the A1, A2, and A3 sites (Table 1).

In the A4 site, eight *I. ricinus* specimens (4 females, 1 male, 3 nymphs) were collected from an area of approximately 147 m^2^ during 15 min. The highest number of active ticks (160 specimens/1 person/1 h) was recorded at a temperature of 24.1 °C and 40.5% RH in the rural recreational area (site B1). The majority of these ticks were *D. reticulatus* specimens.

The number of collected ticks of both species was similar in the sites located in different parts of the city (*I. ricinus* A1 vs. A3; OR = 0.72; 95% IC = 0.33–1.54; *p* = 0.3928; *D. reticulatus* A1 vs. A3; OR = 7.26; 95% IC = 0.89–59.59; *p* = 0.0648) except of localization A2 which was more similar to the rural localization B1 (*I. ricinus* B1 vs. A2; OR = 0.65; 95% CI = 0.24–1.77; *p* = 0.3992; *D. reticulatus* B1 vs. A2; OR = 1.06; 95% CI = 0.64–1.78; *p* = 0.8145). Significantly higher numbers of *D. reticulatus* specimens than *I. ricinus* ticks were found in a suburban recreational area (B1 vs. mean of A1–A3 localizations; OR = 1.98; 95% CI = 1.03–3.79; *p* = 0.0396).

**Figure 2:**
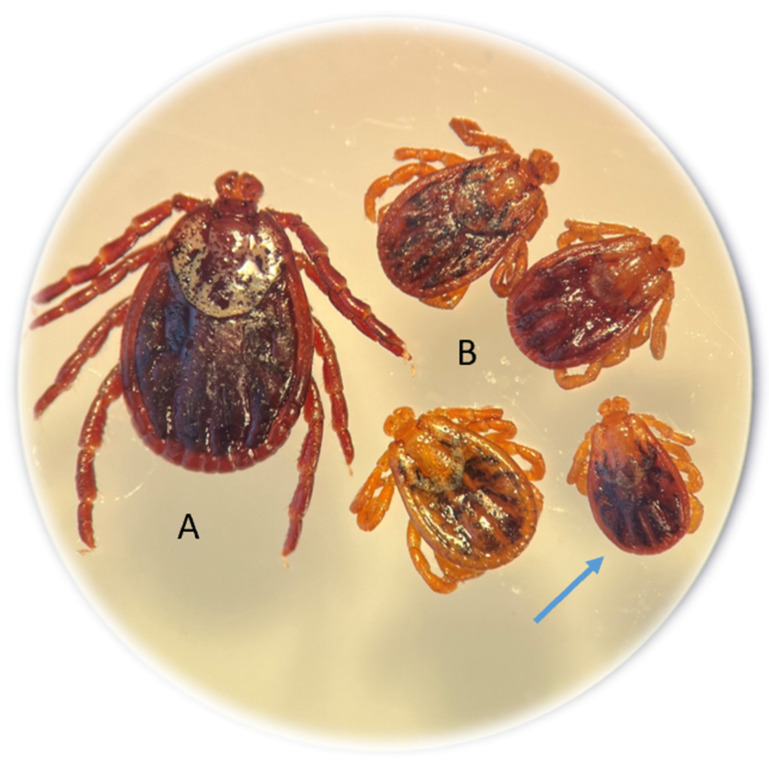
Nanism (dwarfism) in *Dermacentor reticulatus* females collected from vegetation. Typical *D. reticulatus* female (A); dwarf females approximately half the size of a typical female (B). The dimensions of the dwarf female indicated by the arrow with a scale in mm are shown in Figure S2.

Among the 139 adults of this species collected from rural habitats, 7.19% of the specimens (10 females) were dwarf ticks. In the entire study area, abnormal *D. reticulatus* specimens accounted for 5.2% (10/190). The morphological structure of the gnathosoma and idiosoma of the dwarf females was normal (Figure 2).

**Figure 3:**
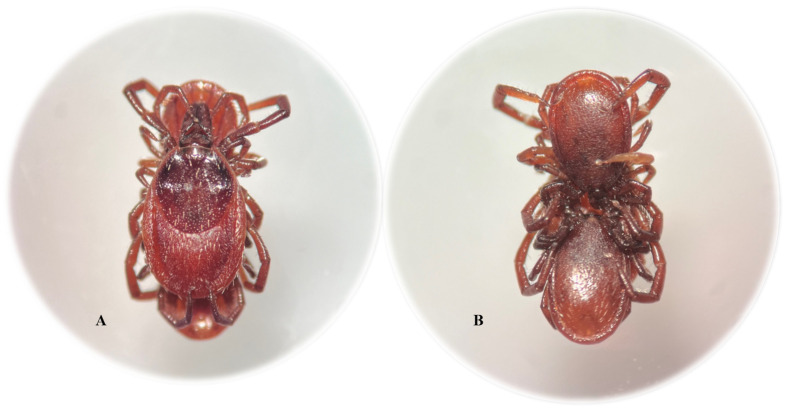
Unusual sexual contact between an *Ixodes ricinus* female and males. Dorsal side of the *I. ricinus* female (**A**); dorsal side of the *I. ricinus* males (**B**).

Ticks *in copula* and specimens exhibiting atypical sexual behavior were observed on plants in January and February. In the A1 site, 7 pairs of *I. ricinus* males and females *in copula* were collected using the flagging method in February. In January, two males copulating simultaneously with one *I. ricinus* female were found among the few active ticks (Figure 3).

The morphological analyses showed that the hypostomes and chelicerae of both *I. ricinus* males were firmly attached to the genital aperture of the *I. ricinus* female. In turn, an *I. ricinus* male in oral-anal contact with a *D. reticulatus* female was found during the field study in the B1 site in February. As in the other cases of sexual contacts between ticks, the hypostome and chelicerae of the *I. ricinus* male were attached to the genital aperture of the *D. reticulatus* female. The mechanical stimuli and the storage of the ticks in 70% ethyl alcohol for over a month until further studies did not cause detachment of the males from the females during their intraspecific or interspecific sexual contacts.

In total, 124 tick specimens were selected from the tick collection (78 *I. ricinus* and 46 *D. reticulatus*) to be tested for the presence of TBPs, i.e., *B. burgdorferi* s.l., *A. phagocytophilum*, *Rickettsia* spp., and *Babesia* spp. The molecular analysis showed the presence of one or two TBPs in 34.62% and 8.41% of the specimens, respectively (Table 2 and Table 3). The DNA of *B. burgdorferi* s.l. spirochetes was identified in 30.77% of the *I. ricinus* specimens, and the DNA of *A. phagocytophilum* was detected in 7.69% of the ticks. Females represented the highest percentage of *I. ricinus* ticks infected with spirochetes at 38.1% (16/42). Mono-infection with *Anaplasma phagocytophilum* was detected only in *I. ricinus* males and was demonstrated in 30.43% (7/23) of the specimens. In turn, in *D. reticulatus*, only mono-infection with *Rickettsia* spp. was shown in 63.0% (29/46) of the studied individuals (Table 2 and Table 3). No genetic material of *Babesia* spp., was identified in the analyzed ticks. None of the pathogens tested were detected in *D. reticulatus* ticks collected in the urban/suburban recreational areas.

## 4. Discussion

It is thought that host-seeking behavior in ticks is driven by temperature to a certain extent [34]. As summarized by Kahl and Gray, little if any activity occurs in *I. ricinus* in the winter in Northern and Central Europe because temperatures are usually too low [62]. The mechanisms regulating *I. ricinus* seasonal activity are determined by a biological strategy involving avoidance of questing in winter when temperatures are too low for efficient host-seeking activity [34,62]. Therefore, the occurrence of progressively warmer winters may result in an increase in the activity of this species during the winter months. Over the last two decades, active ticks of both species have increasingly been found at this time of the year [22,63,64,65,66,67,68,69]. However, unlike *D. reticulatus* [39,65], there is a lack of suitable comparative data for *I. ricinus* from Poland. This study demonstrated, for the first time in Eastern Poland, the mass occurrence of active *I. ricinus* and *D. reticulatus* ticks in one of the winter months (February) and the presence of single *I. ricinus* specimens in January. In our previous studies, *D. reticulatus* specimens had been collected in a meadow ecosystem in the Lublin region in the absence of snow cover at the end of December 2011 and at the beginning of January 2012 (20–42 specimens/1 h of collection) [39]. The abundance of active ticks of both species was similar in the sites located in different parts of the city. However, significantly higher numbers of *D. reticulatus* specimens than *I. ricinus* ticks were found in a suburban recreational area characterized by the occurrence of especially favorable biotic and abiotic conditions for this species.

The presence of as many as 7.19% (10/139) of dwarf *D. reticulatus* females among the adult ticks collected in the rural area may indicate the presence of teratogenic factors in this environment that disrupt tick development but are difficult to identify during field studies. This study describes dwarf *D. reticulatus* specimens collected from plants for the first time. Experimental studies have shown that disruptions in development and morphological changes in juvenile *D. reticulatus* stages can be caused by acaricides used to control plant pests and animal ectoparasites [70,71]. Sublethal concentrations of acaricides (deltamethrin and alphacypermethrin) have been found to disrupt egg development and cause morphological anomalies in *I. ricinus* larvae [72]. Anomalies in ticks can also be induced during embryogenesis by unfavorable humidity levels [73] and cytotoxic substances [74]. Sudden temperature fluctuations during the development of arachnids, including ticks, can be a teratogenic factor as well [75,76,77]. Abnormal *D. reticulatus* specimens collected in their habitat have been very rarely described in the literature to date [78,79]. The type of anomaly detected in the *D. reticulatus* specimens and observed in this study is one of the types of general anomaly referred to as nanism according to the criteria proposed by Campana-Rouget [80].

Interestingly, the field study carried out in February revealed the presence of active host-seeking *D. reticulatus* nymphs, which parasitize small mammals from the families Murinae, Microtinae, and Soricidae but are rarely collected from vegetation even during their seasonal activity in summer [67,81,82]. Endophilic *D. reticulatus* nymphs inhabit rodent burrows and are active for a short period in summer [47]. To date, no questing *D. reticulatus* nymphs have been found in the environment during winter.

Habitat conditions, especially temperature, can change the rhythm of sexual activity of *I. ricinus* adults in winter, which consequently may have an impact on the reproduction of this species and the transmission of tick-borne pathogens. As shown in the present study, in natural conditions, *I. ricinus* males can mate with females as early as February at a temperature of 16.2 °C and 57.2% relative humidity. Similar to other Prostriata representatives, the copulation of *I. ricinus* adults usually takes place on plants but has also been observed on the host [83,84,85,86]. During the present study, atypical sexual behavior of *I. ricinus* and *D. reticulatus* in the natural habitat was detected, for example, simultaneous copulation of two males with an *I. ricinus* female or oral-anal sexual interaction of *I. ricinus* male and *D. reticulatus* female (Figure S3). To the best of our knowledge, such intraspecific contacts between *I. ricinus* ticks have not been reported in the literature to date. Previously, oral-anal interspecific sexual tick interactions were noted only in laboratory conditions [87,88]. Further research is required to identify exogenous factors that stimulate *I. ricinus* and *D. reticulatus* ticks to undertake sexual activity and initiate their atypical sexual behavior in natural conditions. Similarly, little is known about the role of intraspecific and interspecific sexual contacts in the circulation of tick-borne pathogens.

Studies conducted in Northern Hungary (Central Europe) demonstrated that a significant rise in temperature before the peak of spring activity could cause a shift in the activity patterns of *Dermacentor* spp. and *Haemaphysalis inermis* [64]. Reynolds et al. [68] reported similar observations, suggesting that a sudden increase in temperature in late winter (>10 °C in a few days) might lead to an earlier peak in activity of all developmental stages of *I. ricinus*.

During our study conducted in February 2024, historically record-high temperatures ranging from 13.9 °C to 24.1 °C were noted in the collection sites. According to data from the Institute of Meteorology and Water Management—National Research Institute contained in the Bulletins of the State Hydrological and Meteorological Service, the average temperature for February 2024 in the study area was record-breaking (5.6 °C) and many times higher than the average for the 10 preceding years (−0.13 °C) Figure S4. The average annual air temperature in Poland in 2024 was 10.9 °C (deviation from the average for 1991–2020 was as much as 2.2 °C). According to the quantile classification of thermal conditions, 2024 was the warmest year in the history of measurements in Poland in terms of the average temperature. The highest monthly positive temperature anomalies, compared with the norm from 1991 to 2020, occurred in February when the average air temperature at synoptic stations in Poland exceeded the multi-year average by 5.9 °C [89]. The increase in temperature and the associated changes in the hydrological conditions in Eastern Poland may have influenced not only the rhythms of the seasonal activity of ticks but also the development of various stages of these species and may consequently have contributed to the increase in the tick population size.

The presence of potential hosts in the environment [90,91,92,93,94,95] and favorable weather elements interacting with each other, mainly temperature [40,42,96], relative humidity [40,42], or saturation deficit [37,90] are biotic and abiotic factors playing an important role in the proper *I. ricinus* and *D. reticulatus* development cycle, tick density, and phenology.

Following the criteria defined by Supergan and Karbowiak [57], most of the analyzed urban/suburban rural recreation areas can be classified as localities with a high or very high risk of infestation by ticks.

The occurrence of active juvenile and adult stages in the winter months increases the risk of infection of hosts with tick-borne pathogens and, consequently, may support the increased dynamics of pathogen circulation in nature. Climate warming and weather changes also create favorable conditions for the survival and replication of pathogens in the vector, which explains the large increase in human cases of TBDs, e.g., Lyme borreliosis [97]. By their influence on the phenology of tick larvae and nymphs, climatic conditions, especially in winter, may determine the spread of various genotypes of *B. burgdorferi* in hosts [98,99].

The presence of pathogens in tick vectors, such as *B. burgdorferi* spirochetes [100,101,102], *Rickettsia* and *Arsenophonus* bacteria [103,104,105], and tick-borne encephalitis viruses [100,106,107], may modify tick questing behavior, e.g., enhance their locomotor ability. *B. burgdorferi* spirochetes and/or *Rickettsia* spp. can probably initiate interspecies contacts between *I. ricinus* and *D. reticulatus* ticks as well [87,88].

The co-occurrence of active ticks of both species in urban and suburban recreational areas of Eastern Poland in winter not only extends the period of the risk of their attacks on humans and companion animals but also enlarges the spectrum of pathogen species that can be transmitted during tick feeding. The presence of *B. burgdorferi* s.l. spirochetes in as many as 30.77% of *I. ricinus* ticks, including nymphs and adults that were active in February, suggests a high risk of infection of hosts also in the winter season. In recent years, the prevalence of *B. burgdorferi* s.l. and *A. phagocytophilum* in questing *I. ricinus* during the peak seasonal activity in the analyzed region was usually in the range of 15.3–100% [88,108,109] and 1.28–10% [88,110], respectively. In host-seeking *D. reticulatus*, *Rickettsia raoultii* was identified most frequently (43.8–53.0%) [111,112], whereas *B. burgdorferi* (1.6–90%) [88,112] and *Babesia* spp. (2.5–4.5%) were identified less often [112,113,114]. Nevertheless, in habitats with the co-occurrence of both tick species, the percentage of tick infection with some pathogens may be much higher; the prevalence of spirochetes *B. burgdorferi* s.l. and *Rickettsia* spp. may reach even 84.61% and 61.53% in *D. reticulatus* females and 100% and 46.15% in *I. ricinus* males, respectively [88]. In the Lublin macroregion, *B. burgdorferi* (0.7–10.5%) and *A. phagocytophilum* (6.6–8.6%) have been most frequently detected in ticks removed from dogs [115,116].

The high exposure of humans and companion animals to *I. ricinus* and *D. reticulatus* attacks in recreation areas for residents of Eastern Poland is associated not only with the wide distribution of these ticks but also with the rhythms of their diurnal activity. Although the activity of these ticks varies at different times of the day, they can still attack potential hosts throughout the day [117,118].

The occurrence of two species of ticks, *I. ricinus* and *D. reticulatus*, in areas frequently visited by humans and companion animals within the urban agglomeration in Eastern Poland and changes in the host-seeking behavior of these ticks caused by high temperatures in the winter months prompt the need for year-round monitoring of the threat of TBDs and compliance with the recommended principles of tick prophylaxis.

## 5. Conclusions

Climate warming and weather changes may increase the incidence of TBDs in humans and animals by modifying tick behavior and extending the period of tick activity.

Even in the winter months at high temperatures, *I. ricinus* and *D. reticulatus* ticks may appear abundantly in urban and suburban areas of Eastern Poland and pose a high risk of tick attacks on humans and companion animals. The occurrence of *B. burgdorferi* s.l., *Rickettsia* spp., and *A. phagocytophilum* in *I. ricinus*, as well as *Rickettsia* spp. in *D. reticulatus* prompts the need to focus clinical diagnostics on the potential presence of TBD symptoms in patients in this season of the year.

The atypical sexual behavior of ticks in the winter months at extremely high temperatures indicates the need for further research on the impact of these abiotic factors on tick reproduction and the circulation of TBPs in nature. The influence of climatic and environmental changes on the emergence of anomalies in ticks requires clarification as well.

## Figures and Tables

**Table 1:** Number of ticks (Acari: Ixodida) collected from vegetation in recreational areas in Lublin Province (25–27 February 2024).

Collection Site	Weather Factors		Tick Species							
			*Ixodes ricinus*			*Dermacentor* *reticulatus*			Total Tick Number **	Risk of Tick Attack
	Temp. (°C)	RH (%)	F	M	N	F	M	N		
A. Within Lublin County (urban/suburban areas)										
A1 Zalew Zemborzycki Lake *	16.2	57.2	13	12	1	7	5	0	38	high
A2 Stary Gaj forest complex	20.5	44.6	4	1	1	24	9	0	39	high
A 3 green area (field and meadow) in the middle of the Czechów estate	17.6	55.5	8	7	7	1	0	0	23	middle
A4 garden of a private property in the strict city center 147 m^2^	13.9	72.5	4	1	3	0	0	0	8	n.a. ***
B. Rural area										
B 1 Nowy Staw	24.1	40.5	13	2	1	92	47	5	160	very high

* 7 pairs in *copula*, ** per hour of collection/1 person; *** scale of the risk is not applicable; due to the small area, the collection lasted only 15 min; F—females; M—males; N—nymphs; very high risk of tick attack (>50 ticks collected by 1 person/1 h); high risk of tick attack (26–50 ticks collected by 1 person/1 h); middle risk (11–25 ticks collected by 1 person/1 h), based on the scale proposed by Supergan and Karbowiak [57].

**Table 2:** Total number and percentage of ticks infected with *Borrelia burgdorferi* sensu lato, *Anaplasma phagocytophilum*, and *Rickettsia* spp. in the studied areas noted in February 2024 in Eastern Poland.

Tick Species	Developmental Stage	N	1 Pathogen			2 Pathogens	
			*Borrelia burgdorferi* Sensu Lato	*Anaplasma* *phagocytophilum*	*Rickettsia* spp.	*Borrelia burgdorferi* Sensu Lato *+ Rickettsia* spp.	*Borrelia burgdorferi* Sensu Lato *+ Anaplasma phagocytophilum*
*Ixodes ricinus*	Male	23	6 (26.09%)	3 (13.04%)	0 (0.00%)	0 (0.00%)	1 (4.34%)
	Female	42	12 (28.57%)	0 (0.00%)	4 (9.52%)	2 (4.76%)	2 (4.76%)
	Nymph	13	1 (7.69%)	0 (0.00%)	1 (7.69%)	0 (0.00%)	0 (0.00%)
*Dermacentor reticulatus*	Male	14	0 (0.00%)	0 (0.00%)	9 (64.29%)	0 (0.00%)	0 (0.00%)
	Female	32	0 (0.00%)	0 (0.00%)	20 (62.50%)	0 (0.00%)	0 (0.00%)
Total		124	19 (15.32%)	3 (2.42%)	34 (27.42%)	2 (1.61%)	3 (2.42%)

N—number of ticks.

**Table 3:** Total number and percentage of ticks infected with *Borrelia burgdorferi* sensu lato, *Anaplasma phagocytophilum* and *Rickettsia* spp. in the studied areas of Lublin.

Studied Area	Tick Species	Developmental Stage	N	1 Pathogen			2 Pathogens	
				*Borrelia burgdorferi* Sensu Lato	*Anaplasma* *phagocytophilum*	*Rickettsia* spp.	*Borrelia burgdorferi* Sensu Lato *+ Rickettsia* spp.	*Borrelia burgdorferi* Sensu Lato + *Anaplasma phagocytophilum*
A1	*Ixodes ricinus*	Male	12	4 (33.33%)	3 (25.00%)	0 (0.00%)	0 (0.00%)	2 (16.67%)
		Female	13	5 (38.46%)	0 (0.00%)	2 (15.38%)	0 (0.00%)	1 (7.69%)
		Nymph	1	0 (0.00%)	0 (0.00%)	1 (100.00%)	0 (0.00%)	0 (0.00%)
	*Dermacentor reticulatus*	Male	5	0 (0.00%)	0 (0.00%)	3 (60.00%)	0 (0.00%)	0 (0.00%)
		Female	7	0 (0.00%)	0 (0.00%)	4 (57.14%)	0 (0.00%)	0 (0.00%)
A2	*Ixodes ricinus*	Male	1	0 (0.00%)	0 (0.00%)	0 (0.00%)	0 (0.00%)	0 (0.00%)
		Female	4	1 (25.00%)	0 (0.00%)	0 (0.00%)	1 (25.00%)	0 (0.00%)
		Nymph	1	1 (100.00%)	0 (0.00%)	0 (0.00%)	0 (0.00%)	0 (0.00%)
	*Dermacentor reticulatus*	Male	9	0 (0.00%)	0 (0.00%)	6 (66.67%)	0 (0.00%)	0 (0.00%)
		Female	24	0 (0.00%)	0 (0.00%)	16 (66.67%)	0 (0.00%)	0 (0.00%)
A3	*Ixodes ricinus*	Male	7	1 (14.29%)	0 (0.00%)	0 (0.00%)	0 (0.00%)	0 (0.00%)
		Female	8	3 (37.50%)	0 (0.00%)	1 (12.50%)	0 (0.00%)	0 (0.00%)
		Nymph	7	0 (0.00%)	0 (0.00%)	0 (0.00%)	0 (0.00%)	0 (0.00%)
	*Dermacentor reticulatus*	Female	1	0 (0.00%)	0 (0.00%)	0 (0.00%)	0 (0.00%)	0 (0.00%)
A4	*Ixodes ricinus*	Male	1	0 (0.00%)	0 (0.00%)	0 (0.00%)	0 (0.00%)	0 (0.00%)
		Female	4	0 (0.00%)	0 (0.00%)	1 (25.00%)	0 (0.00%)	0 (0.00%)
		Nymph	3	0 (0.00%)	0 (0.00%)	0 (0.00%)	0 (0.00%)	0 (0.00%)
B1	*Ixodes ricinus*	Male	2	1 (50.00%)	0 (0.00%)	0 (0.00%)	0 (0.00%)	0 (0.00%)
		Female	13	3 (23.08%)	0 (0.00%)	0 (0.00%)	1 (7.69%)	0 (0.00%)
		Nymph	1	0 (0.00%)	0 (0.00%)	0 (0.00%)	0 (0.00%)	0 (0.00%)
Total			124	19 (15.32%)	3 (2.42%)	34 (27.42%)	2 (1.61%)	3 (2.42%)

N—number of ticks; A1–A4 urban and suburban recreational areas, B1—rural recreational area.

## Data Availability

The contributions presented in the study are included in the article; further inquiries can be directed to the corresponding authors.

## Supplementary Materials

The following supporting information can be downloaded at: https://www.mdpi.com/article/10.3390/pathogens14040326/s1. Figure S1. Tick collection sites: Zalew Zemborzycki lake (A1); Stary Gaj forest (A2); green area (field and meadow) in the middle of the residential area in the city (A3), private garden at the city center (A4), Nowy Staw site in a rural recreational area located within Kozłowieckie Forests complex (B1) (Fot. Katarzyna Bartosik). Figure S2. Dimensions of the dwarf female *Dermacentor reticulatus* (mm) length (A), width (B) at 32× magnification in a Stemi DV4/DR stereo microscope (Carl Zeiss, Oberkochen, Germany) (Fot.Weronika Buczek). Figure S3. *Ixodes ricinus* male (A) in oral-anal contact with a *Dermacentor reticulatus* female (B). Figure S4. Average area air temperature (AAAT) for January and February in Poland (A); average area air temperature (AAAT) in Poland (B) according to data of the National Hydrological and Meteorological Service https://danepubliczne.imgw.pl/data/dane_pomiarowo_obserwacyjne/Biuletyn_PSHM/, accessed on 15 December 2024. Table S1 Oligonucleotide primers and PCR conditions used in the detection of *Anaplasma phagocytophilum* [59], *Babesia* spp. [60], *Rickettsia* spp. [61], in *Ixodes ricinus* and *Dermacentor reticulatus* ticks.
