# Supplementary material for: Occurrence of Ticks and Tick-Borne Pathogens During Warm Winter—A Snapshot from Central Europe
Source: Pathogens. 2025 Mar 28;14(4):326. doi: 10.3390/pathogens14040326 (PMC12030481; doi:10.3390/pathogens14040326)
Supplement: Supplementary file 1 [file pathogens-14-00326-s001.zip › pathogens-3485348-supplementary.pdf]

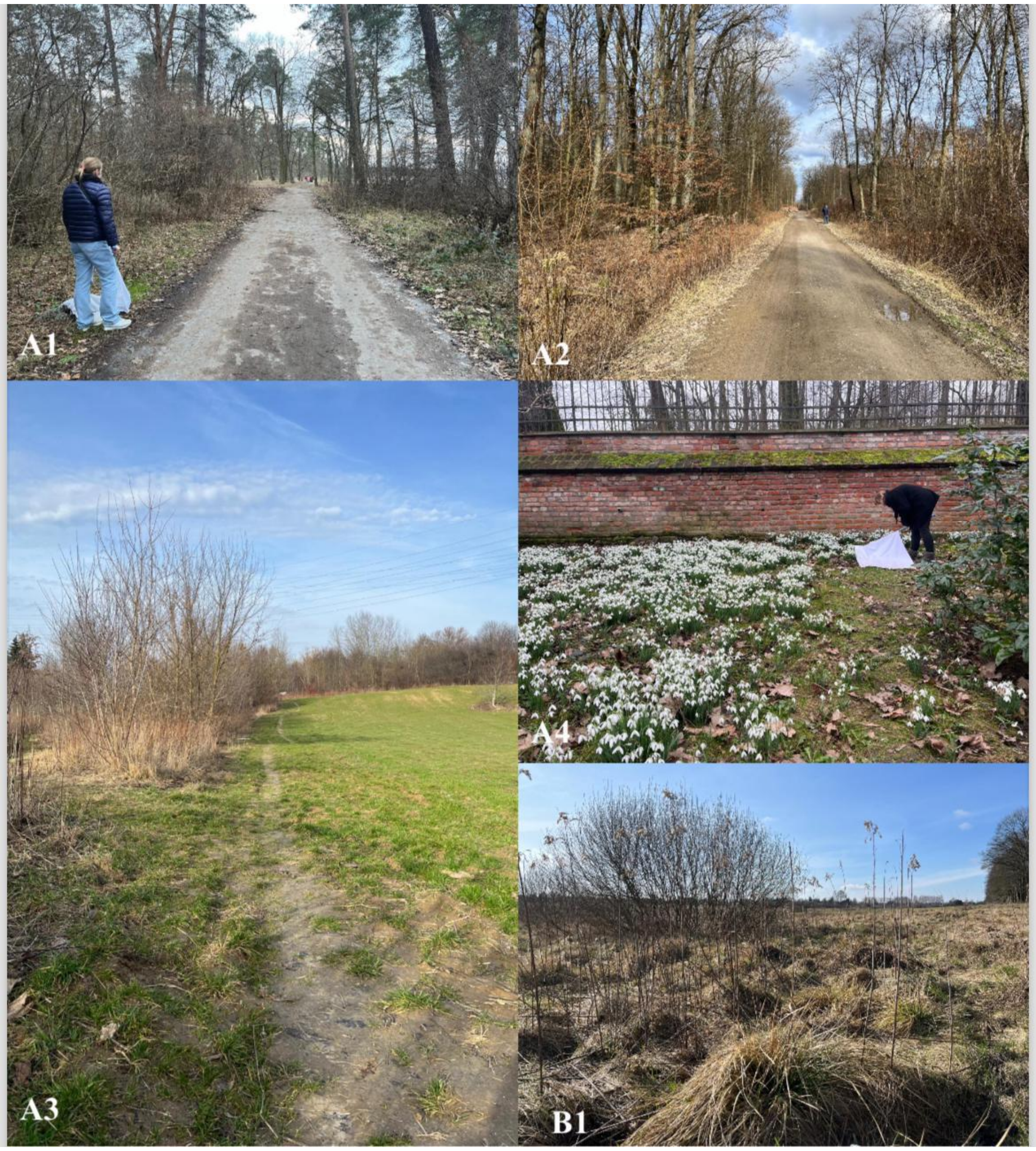

Figure S1. Tick collection sites: Zalew Zemborzycki lake (A1); Stary Gaj forest (A2); green area (field and meadow) in the middle of the residential area in the city (A3), private garden at the city center (A4), Nowy Staw site in a rural recreational area located within Kozłowieckie Forests complex (B1) (Fot. Katarzyna Bartosik).

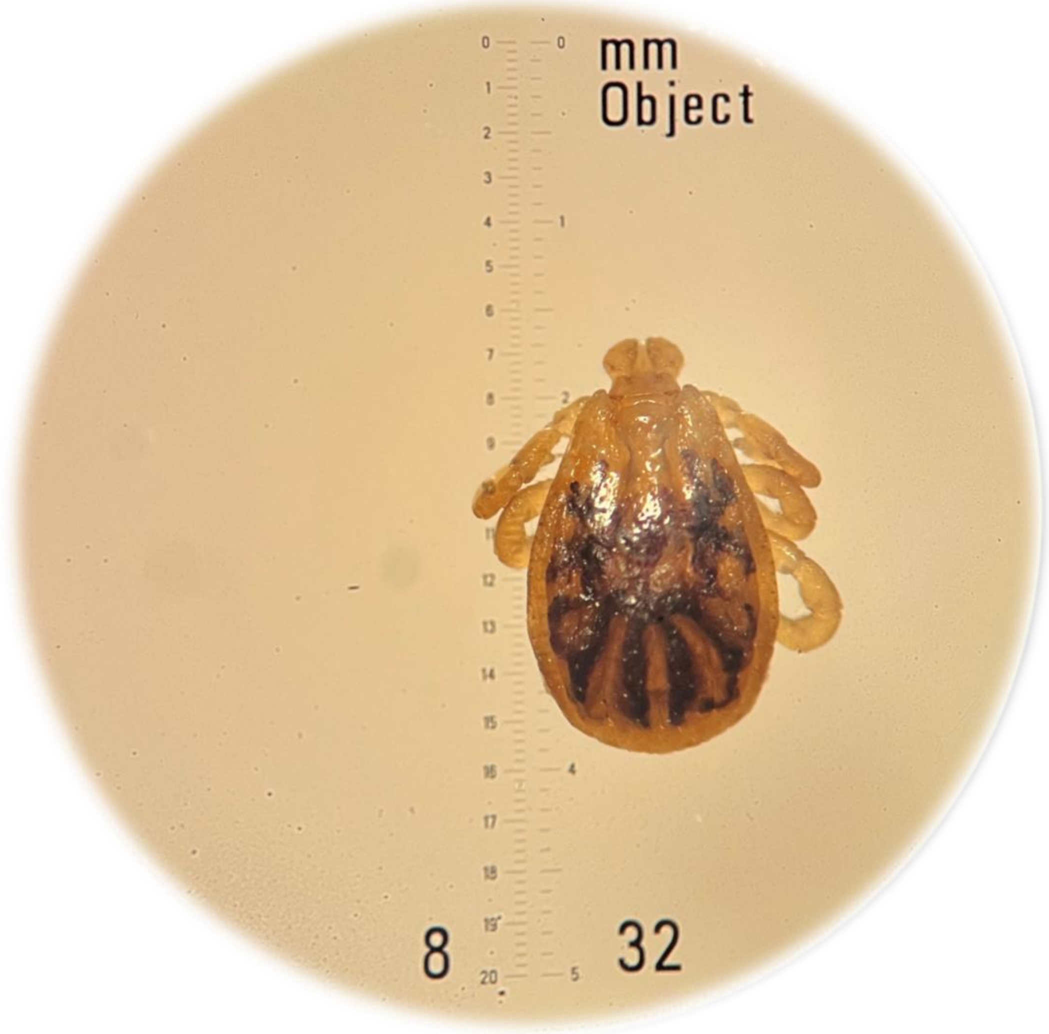

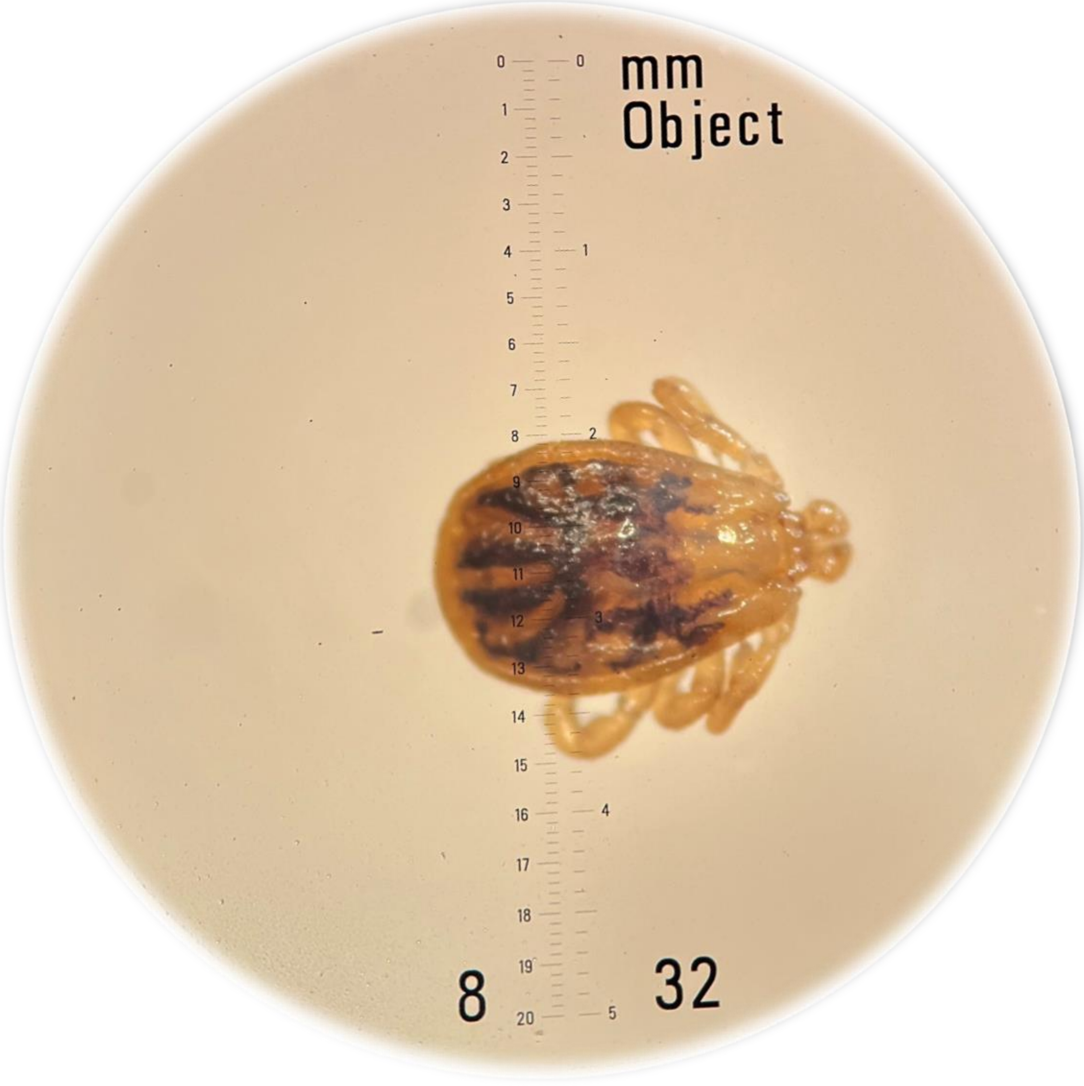

Figure S2. Dimensions of the dwarf female *Dermacentor reticulatus* (mm) length (A), width (B) at 32x magnification in a Stemi DV4/DR stereo microscope (Carl Zeiss, Oberkochen, Germany) (Fot. Weronika Buczek).

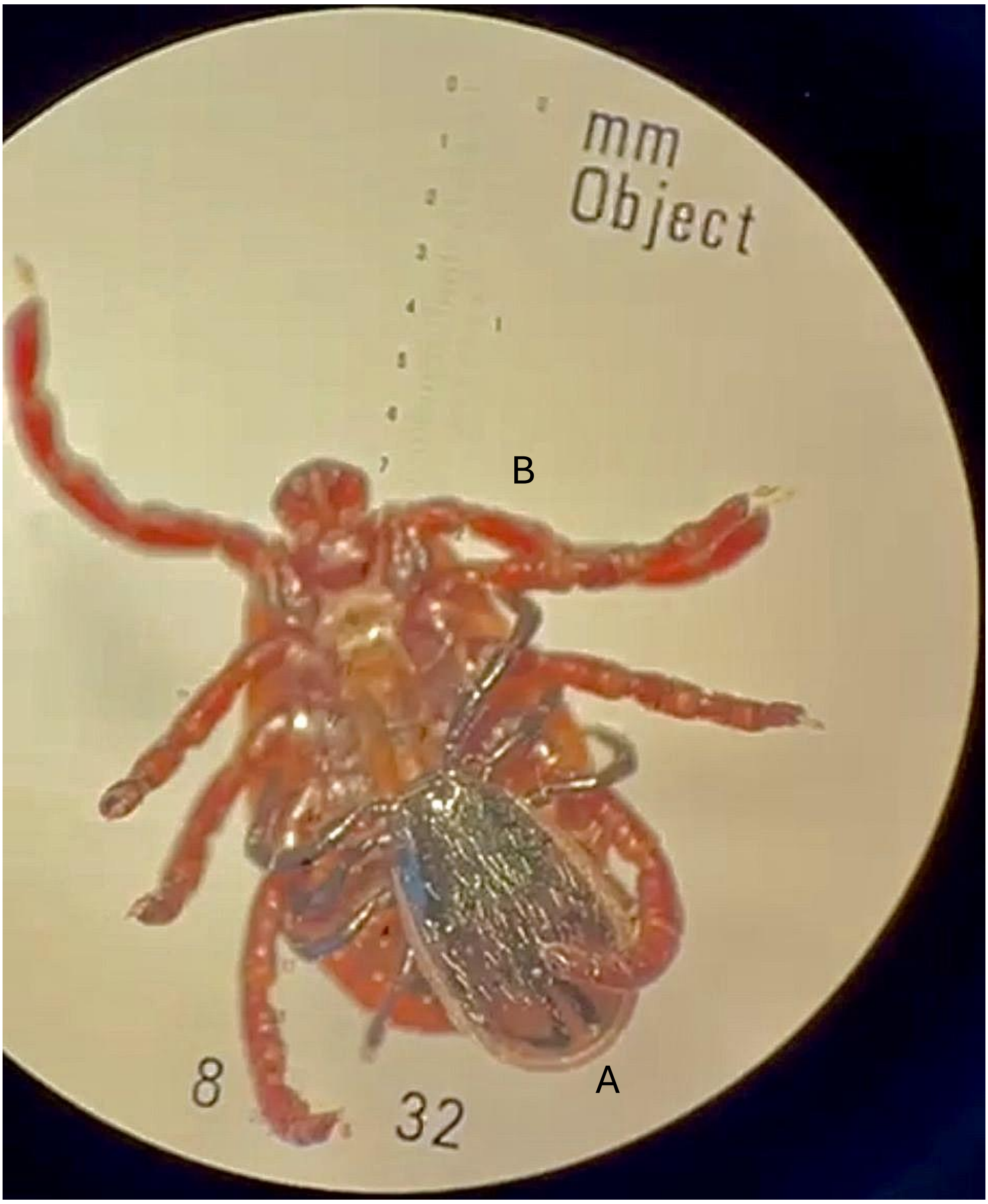

Figure S3. *Ixodes ricinus* male (A) in oral-anal contact with a *Dermacentor reticulatus* female (B).

A

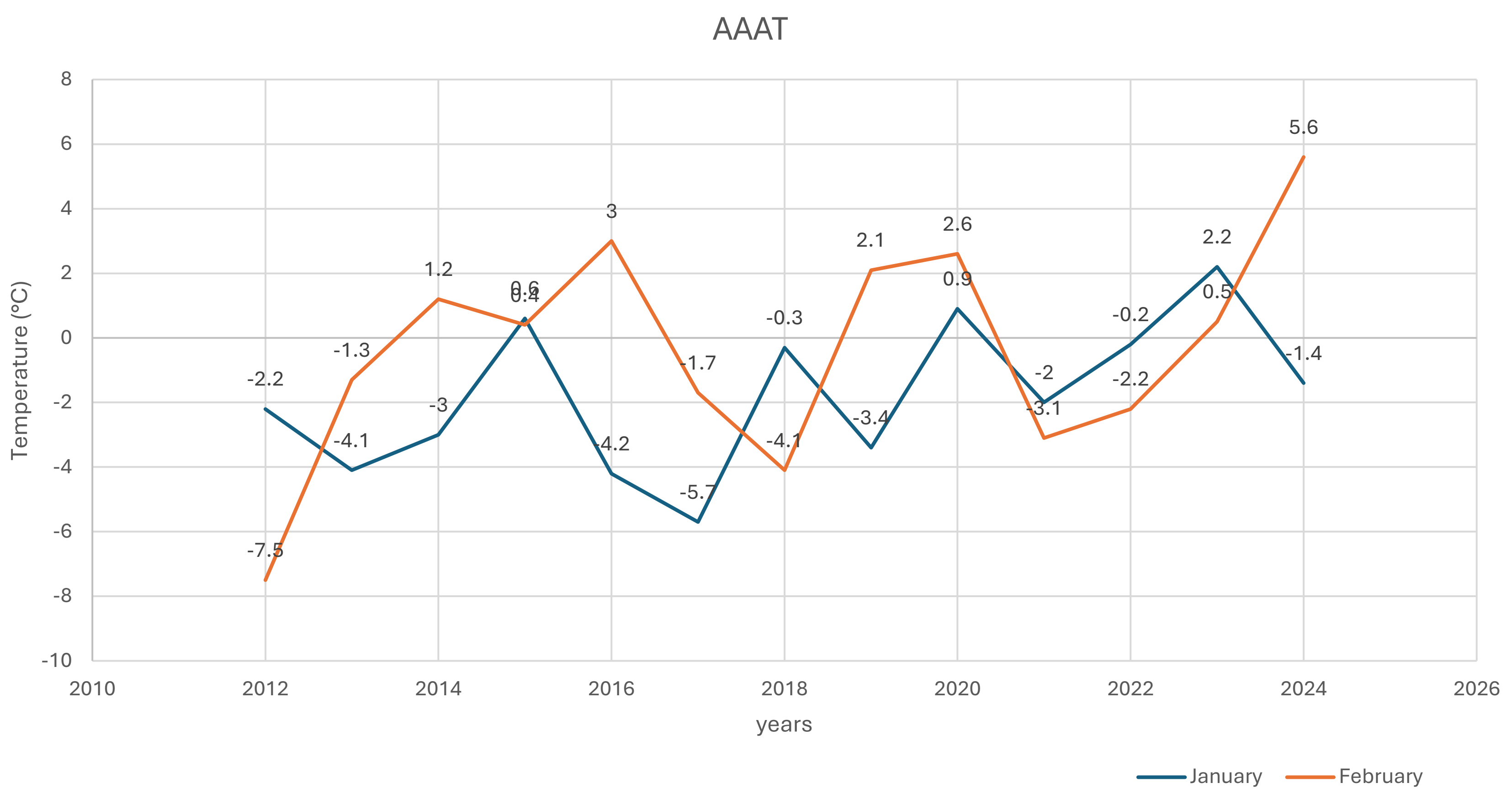

B

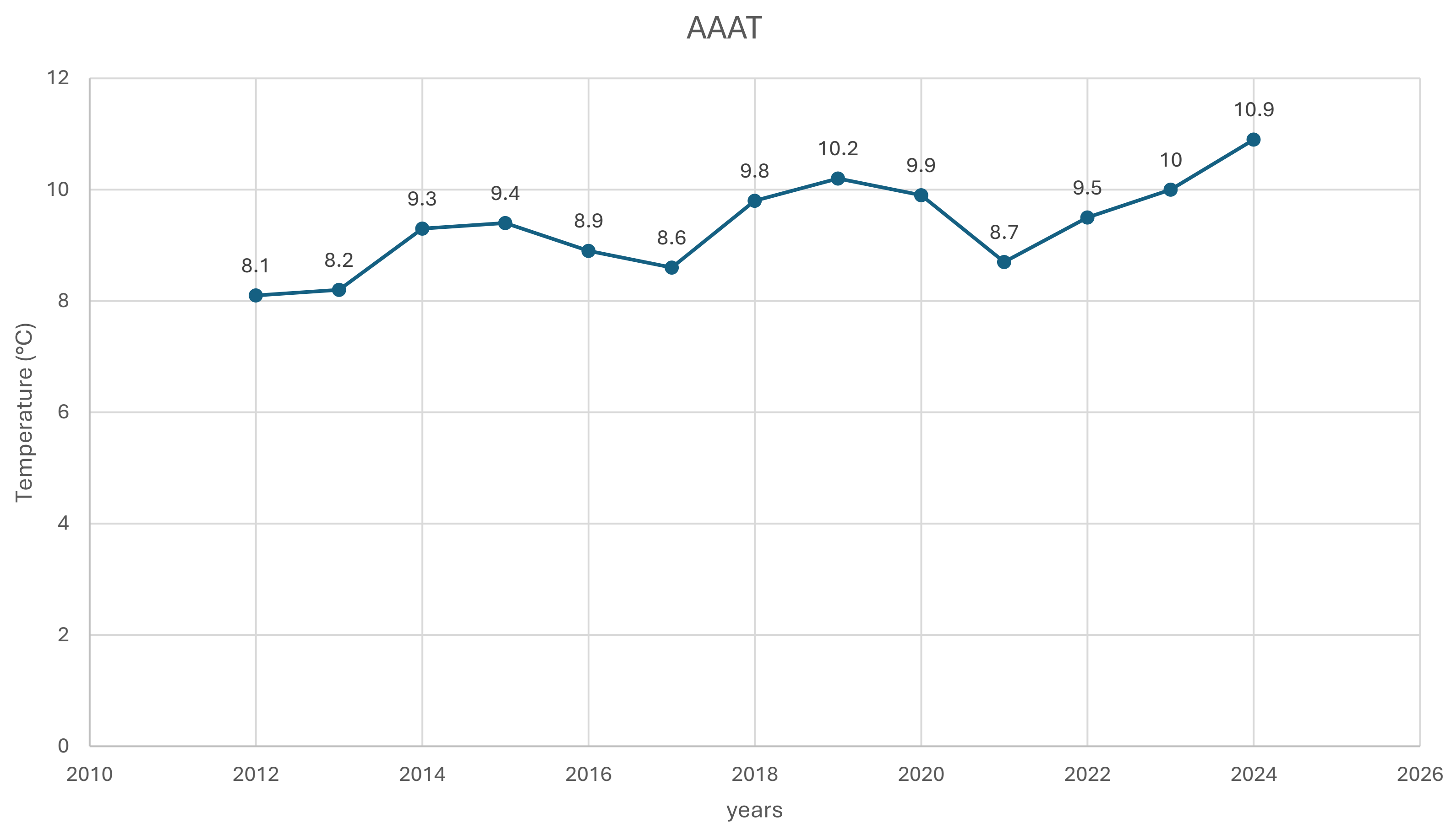

Figure S4. Average area air temperature (AAAT) for January and February in Poland **(A)**; average area air temperature (AAAT) in Poland **(B)** according to data of the National Hydrological and Meteorological Service https://danepubliczne.imgw.pl/data/dane_pomiarowo_obserwacyjne/Biuletyn_PSHM/

Table S1 Oligonucleotide primers and PCR conditions used in the detection of *Anaplasma phagocytophilum* [59], *Babesia* spp. [60]., *Rickettsia* spp. [61], in *Ixodes ricinus* and *Dermacentor reticulatus* ticks.

pathogen	Primer	Sequence (5′-3′)	Gene detected	Size of amplification product [bp]	PCR conditions [°C/s]			No. of cycles
					Denaturation	Annealing	Extension	
Anaplasma phagocytophilum	ge3a	CACATGCAAGTCGAACGGAT TATTC	16S rRNA	932	94/30	55/30	72/60	40
	ge10r	TTCCGTTAAGAAGGATCTAATCTCC						
	ge9f	AACGGATTATTCTTTATAG CTTGCT		546	94/30	55/30	72/60	30
	ge2	GGCAGTATTAAAAGCAGCTCCAGG						
Rickettsia spp.	RpCS.877p	GGGGGCCTGCTCACGGCGG	*gltA*	381	95/20	48/30	60/120	35
	RpCS.1258n	ATTGCAAAAAGTACAGTGAACA						
Babesia spp.	Babfor	GACTAGGGATTGGAGGTC	18S rRNA	620	94/60	53/45	72/90	35
	Babrev	GAATAATTCACCGGATCACTC						
